# Continuous Spinal Anesthesia following Inadvertent Dural Puncture during Epidural Placement for an Emergency Laparotomy

**DOI:** 10.1155/2021/8819864

**Published:** 2021-03-08

**Authors:** Andrew Emyedu, Bernadette Kyoheirwe, Patience Atumanya

**Affiliations:** ^1^Department of Anaesthesia and Critical Care, Makerere University, College of Health Sciences, P.O. Box 7062, Kampala, Uganda; ^2^Department of Anaesthesia, Uganda Cancer Institute, P.O. Box 3935, Kampala, Uganda

## Abstract

*Summary*. Emergency exploratory laparotomy conducted under continuous spinal anesthesia using a standard epidural set following an accidental dural puncture. *Background and Objectives*. Continuous spinal anesthesia is one of the least utilized regional anesthesia techniques globally. It could be an alternative anesthesia technique for abdominal and lower limb surgeries following an accidental dural puncture. The aim of this report was to describe a case in which continuous spinal anesthesia was successfully conducted for emergency exploratory laparotomy following an accidental dural puncture during epidural placement. *Case Report*. A 38-year-old male presented to our accident and emergency unit with a one-day history of colicky abdominal pain associated with constipation, abdominal distension, and vomiting. He was diagnosed with intestinal obstruction and underwent an emergency exploratory laparotomy under continuous spinal anesthesia using a standard epidural set following an accidental dural puncture. *Conclusion*. This case demonstrates that in case of an accidental dural puncture during epidural placement, the catheter can be advanced into the intrathecal space and continuous spinal anesthesia conducted for abdominal surgeries using a standard epidural catheter.

## 1. Introduction

Epidural analgesia is one of the most studied postoperative pain management modalities for thoracic, abdominal, and lowerlimb surgeries; however, epidural placement can be complicated by accidental dural puncture (ADP) which occurs more commonly among junior trainees [1]. The management options following ADP include placement of the epidural at a proximal intervertebral interspace, conversion to single-shot spinal anaesthesia, general anesthesia, or abandoning the procedure [2]. Continuous spinal anesthesia (CSA) using an epidural catheter provides an alternative anesthesia technique, and if the epidural catheter is left in place for over 24 hours, it reduces the occurrence of postdural puncture headache (PDPH) [2–4]. In this report, we describe a case in which CSA was successfully conducted using a standard epidural catheter for an emergency exploratory laparotomy following inadvertent dural puncture.

## 2. Case Report

A 38-year-old male, body mass index of 26.4, American Society of Anesthesiologists (ASA) physical status 1E, presented to the accident and emergency unit at a public University-affiliated hospital with a one-day history of colicky abdominal pain associated with constipation, abdominal distention, and bilious vomiting. His past surgical history included an uneventful exploratory laparotomy for intestinal obstruction 13 years prior to this episode. Initial examination showed a middle-aged well-nourished male, with mild pallor of the mucous membranes, moderate dehydration, and tachypnea. The abdomen was distended with peritoneal signs. The investigations revealed a normal complete blood count with a hemoglobin of 11.9 g/dl. The abdominal ultrasound showed distended bowel loops suggestive of intestinal obstruction, and he consented for an emergency exploratory laparotomy under general anesthesia and a neuraxial (epidural) analgesic plan.

After establishing peripheral venous access and placement of monitors (pulse oximetry, noninvasive blood pressure monitoring, and 3-lead electrocardiography), the patient was premedicated with ceftriaxone 2 g, metronidazole 500 mg, metoclopramide 10 mg, ondansetron 8 mg, and glycopyrrolate 0.2 mg. A sterile field was established, and after local infiltration with 3 ml of 2% lidocaine, an 18G Tuohy needle (Runquiang, Zhejiang, China) was used to locate the epidural space at the T10/11 vertebral intespace using a midline approach. A loss of resistance was obtained at 5 cm; however, we noted free-flowing cerebral spinal fluid (CSF) at the needle hub. The needle was occluded and kept in place. The patient was informed, and verbal consent obtained to advance the catheter into the intrathecal space for continuous spinal anesthesia (CSA).

**Figure 1:**
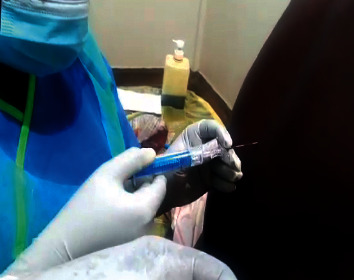
A wet tap during epidural placement.

**Figure 2:**
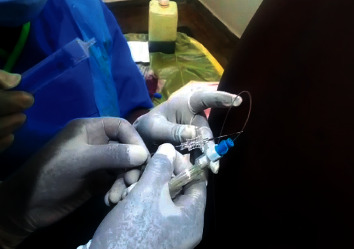
Threading catheter into the intrathecal space.

The 20G multiorifice catheter and filter were primed with 2 ml of 0.5% isobaric bupivacaine and advanced 3 cm into the intrathecal space. The patient did not complain of paresthesia during needle or catheter insertion, and the catheter was secured in place using a transparent sterile tape, as shown in Figures 1 and 2. The patient reported difficulty in breathing when in supine position due to the abdominal distension; however, this was relieved upon activation of the intrathecal catheter. A total of 10 mg of isobaric bupivacaine (administered in 5 mg boluses) was used to achieve adequate sensory blockade (T4 to L2).

**Figure 3:**
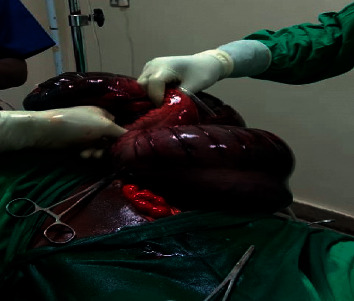
Gangrenous bowel discovered during exploratory laparotomy.

He scored one on the modified Bromage score (could flex the hip) and zero on the Epidural Scoring Scale for Arm Movement (ESSAM) score (normal handgrip, flexion at the elbow and wrist). He received 100 mcg of intrathecal morphine (ITM) and 5 mg of isobaric bupivacaine top-up to maintain adequate sensory blockade throughout the procedure. We had one episode of hypotension (30% drop from baseline mean arterial pressure) after the topup dose of bupivacaine which was managed using an adrenaline infusion (300 mcg of adrenaline in 500 mls of Ringer's lactate) titrated to maintain mean arterial pressure (MAP) above 65 mmHg. The procedure lasted 145 minutes during which the patient received supplemental oxygen through nasal prongs, a total of 1500 ml of lactated Ringers' solution, and 180 ml of O-negative blood. He was diagnosed with gangrenous gut secondary to sigmoid volvulus. The patient had approximately 35 cm of the sigmoid colon resected, and a colostomy placed in the left iliac fossa, Figure 3.

The patient was transferred to the postanesthesia care unit hemodynamically stable and then to the ward. Postoperative pain was managed with parenteral paracetamol 1g eight hourly and, at 24 hours, received an additional 100 ug of ITM after which the catheter was removed. Postoperative pain was assessed using the numerical rating scale and was 0/10 at 12 hours and 2/10 at 24 and 72 hours.

The patient reported no episodes of fever, nausea, vomiting, pruritus, urine retention, or postdural puncture headache (PDPH). He was discharged from the hospital on the third postoperative day and reported no neurologic sequelae such as paresthesia or paraplegia during the 21-day weekly follow-up. The surgeons and the patient were satisfied with the anesthetic technique and pain control in the immediate postoperative period.

## 3. Discussion

We present a case in which a standard 18G epidural set was successfully used to conduct continuous spinal anesthesia (CSA) for a patient undergoing an emergency exploratory laparotomy following an accidental dural puncture (ADP).

Single-shot spinal anesthesia has limited application in abdominal surgery due to the fast rate of regression of sensory blockade and unpredictable hemodynamic derangements [5]. Continuous spinal anaesthesia provides an alternative anesthetic technique for these surgeries [6]. Various advantages have been reported including the ability to titrate the level of sensory blockade using low doses of local anesthetics (LA), fast onset of the block, flexible duration of anesthesia, and reduced incidence of LA systemic toxicity [5, 7, 8].

Historically, CSA was reserved for the geriatric patients and unstable cardiac patients undergoing noncardiac surgery [5, 7, 9, 10] but is now more widely used for patients undergoing lower limb surgeries, mastectomies, urology procedures, and more recently, obstetric and gynecological procedures including labor analgesia [4, 6, 8, 11, 12].

Despite the convincing safety evidence for CSA, it has not gained popularity among anesthesia providers due to scarcity of the CSA sets especially in resource-constrained settings such as ours [12], difficult intrathecal catheter insertion despite adequate CSF flow and fear of neurologic complications like cauda equina syndrome (CES) [13]. Cauda equina syndrome was thought to result from either maldistribution of the local anesthetic following slow injection through microcatheters or use of high concentrations of the local anesthetics [6, 13]. However, Lux and colleagues reported no neurologic sequelae among 1212 patients in whom microcatheters were used; therefore, the most plausible cause of CES is use of high concentrations of local anesthetics like 5% lidocaine, which was subsequently phased out [6].

PDPH is one of the most frequently occuring neurological complications following an accidental dural puncture (ADP) and is thought to result from CSF leakage through the hole created in the dura [2]. Advancing the catheter into the intrathecal space and leaving it in situ for 24 hours has been shown to reduce the incidence of PDPH [3]. The postulated mechanism of action is it acts as a mechanical block preventing CSF leakage through the hole created in dura and alsocauses early inflammation at the dural puncture site which further reduces CSF leakage [4] and if intrathecal morphine is administered through the catheter (as was done in this case), its analgesic effects would further reduce the incidence of PDPH [2, 4].

During the surgery, we had one significant episode of hypotension which warranted immediate attention, and this has been a rare occurrence in similar studies [5]. This could be explained by the fact that we used a higher dose of LA to redose the catheter (5 mg) as opposed to the more studied 2.5 mg boluses [2, 5, 7].

When used appropriately, CSA provides adequate postoperative pain management superior to abdominal wall blocks and is comparable to epidural analgesia (which is a gold standard following abdominal surgeries) [14, 15]. Similar to other resource-constrained settings where cost implications play a significant role in the analgesic technique utilized, use of CSA offers a cost-effective benefit over abdominal wall blocks that use higher drug volumes and doses and may require continuous nerve block needles and elastomeric pumps which are also expensive and not easily accessible.

## 4. Conclusion

Continuous spinal anesthesia avoids the complications associated with general anesthesia while maximizing the benefits of both epidural and single-shot spinal anesthesia like fast onset of anesthesia and ability to prolong the duration of anesthesia, for long surgeries.

This is the first case report in our setting in which continuous spinal anesthesia was conducted following accidental dural puncture; more studies are required to generate a pool of evidence before its routine use in our center.

## Data Availability

This is a case report with no extra datasets, and all the informationis available within the article. However, some literature studies supporting our findings are Ozturk E, Gokce M, Gunaydin B, Babacan A. Continuous spinal anesthesia after unintentional dural puncture during attempted epidural anesthesia for mastectomy. Regional anesthesia and pain medicine. 2004; 29(4):381. 2. Chaudhary K, Saxena KN, Taneja B, Gaba P, Anand R. Intrathecal catheterisation for accidental dural puncture: A successful strategy for reducing post-dural puncture headache. Indian journal of anesthesia. 2014; 58(4):473. Parthasarathy S, Ravishankar M. Continuous spinal anesthesia with epidural catheters: An experience in the periphery. Anaesthesia, Essays and Researches. 2011; 5(2):187.
